# Chromosomes of *Belonocnema treatae* Mayr, 1881 (Hymenoptera, Cynipidae)

**DOI:** 10.3897/CompCytogen.v9i2.6534

**Published:** 2015-05-26

**Authors:** Vladimir E. Gokhman, James R. Ott, Scott P. Egan

**Affiliations:** 1 Botanical Garden, Moscow State University, Moscow 119234, Russia; 2 Population and Conservation Biology Program, Department of Biology, Texas State University, San Marcos, TX 78666, USA; 3 Department of BioSciences, Rice University, Houston, TX 77005, USA

**Keywords:** Hymenoptera, Cynipidae, *Belonocnema treatae*, gall wasps, chromosomes, karyotype

## Abstract

Chromosomes of the asexual and sexual generation of the gall wasp *Belonocnema treatae* Mayr, 1881 (Cynipidae) were analyzed. Females of both generations have 2n = 20, whereas males of the sexual generation have n = 10. Cyclical deuterotoky is therefore confirmed in this species. All chromosomes are acrocentric and form a continuous gradation in size. This karyotype structure is probably ancestral for many gall wasps and perhaps for the family Cynipidae in general. Chromosome no. 7 carries a characteristic achromatic gap that appears to represent a nucleolus organizing region.

## Introduction

Parasitic Hymenoptera are one of the largest, taxonomically complicated and economically important insect groups (Rasnitsyn 1980, Heraty et al. 2011). The overwhelming majority of this group attacks insects and some other arthropods; however, certain taxa of the ‘parasitic’ Hymenoptera are in fact secondarily phytophagous (Quicke 1997). Among these taxa, gall wasps of the family Cynipidae are the most diverse, with their world fauna exceeding 1300 species (Ronquist 1999, Abe et al. 2007, Liljeblad et al. 2011). Many gall wasps exhibit cyclical parthenogenesis, i.e. they have heterogonous life cycles with temporally segregated sexual and asexual generations (Crozier 1975, Stone et al. 2002). The cynipid, *Belonocnema treatae* Mayr, 1881 induces galls on live oaks (*Quercus* spp.) in the series *Virentes* (Muller 1961, Melika and Abrahamson 2002). In the Edwards Plateau region of central Texas, USA, both generations are host specific to *Quercus fusiformis* Small (Lund et al. 1998). The asexual generation of *Belonocnema treatae* develops within single-chambered, spherical galls on the undersides of leaves during the summer and fall and emerges in the fall and winter, whereas the sexual generation develops within multi-chambered galls on the roots, and males and females emerge during the spring (Lund et al. 1998).

Chromosomes of more than twenty species of the family Cynipidae have now been studied (see Gokhman 2009 for review). Karyotypes of many cynipid gall wasps exhibit a relatively high degree of similarity. Indeed, most genera and species have the same chromosome number, n = 10 (Sanderson 1988). Nevertheless, all four studied members of the genus *Diplolepis* Fourcroy, 1785 show another n value, i.e. n = 9. Moreover, chromosome sets with deviating karyotype structure have been detected within the genus *Andricus* Hartig, 1840 (Abe 1998, 2007). In this genus, the majority of species also have n = 10, although a few closely related taxa have chromosome sets with n = 6 and 5. Furthermore, the latter karyotypes belong to a particular species complex where cryptic species were discovered (Abe 1998). Interestingly, similar chromosome numbers, n = 10 and 9, are characteristic of five studied species of another cynipoid family, Figitidae, in which two other species with n = 11 and 5 were also found (Gokhman 2009, Gokhman et al. 2011).

Recent observations reported by Hjelmen et al. (2013) suggest that observed values for the genome size of male and asexual female *Belonocnema treatae* differ from values expected from haplo-diploidy. We have undertaken the present study to investigate chromosomes of this species and to determine whether variation in karyotype structure is present within and/or between the asexual and sexual generations of *Belonocnema treatae* within a single population.

## Material and methods

Samples of the asexual and sexual generations of *Belonocnema treatae* developing within galls on *Quercus fusiformis* from central Texas, USA, were collected near San Marcos (Texas) and husbanded in the lab during September 2013 and March 2014 respectively. Prepupae and early pupae of *Belonocnema treatae* were extracted from the dissected galls. Chromosomal preparations were obtained from developing ovaries and, in case of males, prepupal cerebral ganglia following the protocol provided by Imai et al. (1988) with some modifications. Mitotic divisions were studied and photographed using an optic microscope Zeiss Axioskop 40 FL fitted with a digital camera AxioCam MRc. To obtain karyograms, the resulting images were processed with image analysis programs Zeiss AxioVision version 3.1 and Adobe Photoshop version 8.0. Mitotic chromosomes were measured on thirty haploid metaphase plates using Adobe Photoshop and then classified according to the guidelines provided by Levan et al. (1964).

## Results

**Figure 1.**
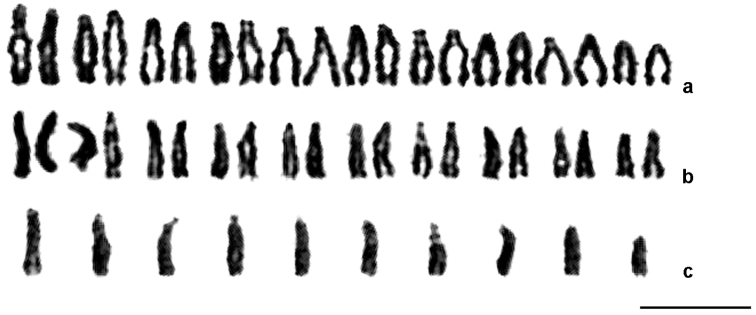
Karyograms of *Belonocnema treatae*. **a** asexual female **b** sexual female **c** male. Bar = 10 μm.

Mitotic metaphase plates from eleven females of the asexual generation as well as six females and five males of the sexual generation of *Belonocnema treatae* were analyzed. Females of both the asexual and sexual generations have identical karyotypes with 2n = 20 (Fig. 1a, b), whereas males of the sexual generation have n = 10 (Fig. 1c). All chromosomes form a continuous gradation in size (perhaps except for the smallest chromosome; Table 1) and are clearly acrocentric, although shorter arms are visible in many elements. No aneuploid specimens or individuals with other unusual karyotypic features were found. Chromosome no. 7 carries a characteristic achromatic gap in the longer arm near the centromere (Fig. 1a–c). This gap appears to represent a nucleolus organizing region (NOR) and is best visible in the male karyotype, possibly because of the stronger spiralization of the chromosomes.

**Table 1.** Relative lengths (RL) of *Belonocnema treatae* chromosomes from haploid metaphase plates (mean ± SD).

Chromosome no.	RL
1	11.91 ± 0.52
2	11.34 ± 0.37
3	10.89 ± 0.36
4	10.44 ± 0.28
5	10.19 ± 0.21
6	9.88 ± 0.30
7	9.65 ± 0.28
8	9.35 ± 0.30
9	8.90 ± 0.41
10	7.45 ± 0.62

## Discussion

Our results show that *Belonocnema treatae* exhibits cyclical deuterotoky, similarly to many other members of the family Cynipidae studied in this respect (reviewed in Crozier 1975 and Stone et al. 2002). The chromosome number found in *Belonocnema treatae*, i.e. n = 10 (2n = 20), is the most common in the family. Moreover, all chromosomes of this species appeared to be acrocentric. Despite karyotypes of most members of the Cynipidae containing at least some biarmed chromosomes (see e.g. Sanderson 1988), only acrocentrics were found in the chromosome set of another species, i.e. *Dryocosmus kuriphilus* Yasumatsu, 1951 (Abe 1994). Interestingly, both *Dryocosmus* Giraud, 1859 and *Belonocnema* Mayr, 1881 represent the least advanced lineages within their clades, i.e. within the *Neuroterus*-group and *Cynips*-group respectively (see Tree 7 in Liljeblad et al. 2008), and therefore this karyotype structure is likely to be ancestral for members of their common clade within the tribe Cynipini, and perhaps for the family Cynipidae in general. However, biarmed chromosomes apparently predominate in the karyotype of *Callirhytis quercuspomiformis* (Bassett, 1881) with n = 10 (Goodpasture 1975). Since this species is the only studied member of *Callirhytis* Förster, 1869 (in turn, the least advanced examined genus of Cynipini), we cannot exclude the presence of metacentrics and/or submetacentrics within the ancestral karyotype of the above-mentioned tribe/family.

Although certain communications claimed that *Belonocnema treatae* possessed a special sex determination mechanism, these reports were mainly based on putative differences in the genome size between various populations and generations of this species (see e.g. Hjelmen et al. 2013). However, recent studies suggest that these results could be affected by tannins coming from the galls (Hjelmen et al. 2014).

The present study has also revealed a single achromatic gap (presumably NOR) in the haploid karyotype of *Belonocnema treatae*. Among other Cynipidae, similar results were obtained in the only species studied in this respect, *Diplolepis rosae* (Linnaeus, 1758) using FISH with 18S rDNA probe (Gokhman et al. 2014).
